# Management of Complex Peri-Prosthetic Joint Infection Following Total Knee Arthroplasty with Soft Tissue Defects: Case Series and Multidisciplinary Approach

**DOI:** 10.3390/jpm16050238

**Published:** 2026-04-30

**Authors:** Katelynn Murray Whelan, Gerard Anthony Sheridan, Kenneth Joyce, Alan Hussey, Jason S. Hoellwarth, Justina Baltrunaite

**Affiliations:** 1 Department of Orthopaedic Surgery, University Hospital Galway, H91 YR71 Galway, Ireland; 2 Department of Plastic & Reconstructive Surgery, University Hospital Galway, H91 YR71 Galway, Ireland; 3 Hospital for Special Surgery, New York, NY 10021, USA

**Keywords:** total knee arthroplasty, peri-prosthetic joint infection, soft tissue reconstruction, antibiotic spacer, individualized approach, soft tissue complications

## Abstract

**Background:** Peri-prosthetic joint infection (PJI) following total knee arthroplasty complicated by soft tissue compromise presents a major reconstructive challenge. Successful management requires the eradication of infection while restoring durable soft tissue coverage and limb function. This study reports the outcomes of a patient-specific, multidisciplinary orthoplastic approach to complex knee PJI. **Methods:** We retrospectively reviewed five patients with complex infected knee arthroplasty and associated soft tissue compromise managed at our institution between 2021 and 2025 by a single orthopaedic surgeon and two plastic reconstructive surgeons. All cases required personalized management, including the use of custom spacers, patient-specific orthopaedic reconstruction, and individualized soft tissue reconstruction techniques. Data collected included patient demographics, infection characteristics, reconstructive techniques, and functional outcomes. **Results:** All patients achieved durable soft tissue coverage and infection eradication at final follow-up. Of the five patients, one underwent primary closure of a persistent sinus, one required a local axial bi-pedicled flap for sinus control and soft tissue closure, two were managed with medial gastrocnemius flaps, and one complex case with an associated bone defect required a custom-designed spacer to achieve stability and dead-space management. **Conclusions:** In this retrospective case series, we aim to demonstrate that complex knee PJI with associated soft tissue defects may be successfully managed with an individualized, multidisciplinary strategy. We aim to demonstrate the feasibility of such an approach in a tertiary referral centre and to highlight the importance of customisation in achieving infection control and limb preservation.

## 1. Introduction:

Peri-prosthetic joint infection (PJI) remains one of the most devastating complications following total knee arthroplasty (TKA), with a reported incidence of 0.5–2% after primary arthroplasty and up to 3–10% following revision procedures in the United States [1,2,3,4]. PJI can be difficult to diagnose and has no universally accepted reference standard definition. Despite being difficult to describe definitively, the European Bone and Joint Infection Society has recently classified PJI as the presence of a sinus tract or two positive identical cultures as evidence of infection [5]. Contemporary epidemiological data demonstrate a persistent increase in PJI incidence, driven by rising arthroplasty volumes and an ageing, increasingly comorbid patient population. PJI is now one of the leading indications for revision TKA worldwide [1,6]. PJI is associated with significant morbidity, prolonged hospitalisation, repeated surgical interventions, and substantial healthcare costs, making it a major clinical and socioeconomic burden [1,7]. PJI management is further complicated when accompanied by soft tissue compromise, which can present with sinus tract formation, wound dehiscence, or large soft tissue defects [8]. Soft tissue compromise in the context of PJI represents a critical determinant of outcome. Soft tissue compromise increases the clinical complexity exponentially, as it is significantly challenging to balance achieving infection control and providing stable coverage, while also maintaining limb function [8,9]. In the case of complex infected knee arthroplasty with associated soft tissue defects, the traditional treatment algorithms may be insufficient, as many of these traditional management strategies often fail to account for the variability of defect size, bone loss, and local tissue quality. These standardised protocols rarely address the combined demands of infection eradication, mechanical stability, and soft tissue reconstruction in complex cases [1,10]. Conventional treatment algorithms frequently assume adequate soft tissue coverage, an assumption that does not hold true in complex revision cases [4,8]. Although the use of treatment algorithms available today yields overall acceptable success rates, optimal approaches to treatment have not yet been accurately identified, suggesting a requirement for an individualized management approach. As such, patient-specific orthoplastic strategies integrating orthopaedic, plastic surgical, and infectious disease expertise are increasingly advocated [1,3,11].

While there is evidence surrounding the use of muscle flaps, such as the medial gastrocnemius, in achieving durable coverage, there remains limited evidence on the integration of such reconstructive strategies with the use of custom-made antibiotic spacers to simultaneously address bone loss, dead space, and soft tissue deficits. This gap in the literature underscores the need for a multidisciplinary approach that combines orthopaedic reconstruction with tailored plastic surgical reconstructive techniques. By adapting spacer design, antibiotic treatment, and flap selection to each patient’s clinical status, clinicians may achieve better infection control and functional preservation compared with a uniform algorithmic treatment approach. We aim to describe a tailored, multidisciplinary approach integrating orthopaedic reconstruction, customised antibiotic spacers, and tailored soft tissue reconstruction for complex infected TKA, providing transparent methodology and reproducible outcomes. This study evaluated outcomes in patients with complex infected knee arthroplasty and associated soft tissue defects managed with a patient-specific strategy in a retrospective cohort.

## 2. Method

This study was designed as a retrospective case series conducted at a tertiary referral centre of University Hospital Galway for complex orthopaedic and plastic reconstructive surgery. The study included patients who underwent management for infected total knee arthroplasty (TKA) complicated by soft tissue defects between January 2021 and September 2025. A total of five patients who underwent surgery for infected knee arthroplasty associated with soft tissue defects were included. The study was conducted in accordance with the Declaration of Helsinki and approved by the Clinical Research Ethics Committee of University Hospital Galway (protocol code C.A.3616 and date of approval 27 March 2025). Written informed consent was obtained from all patients for publication of anonymised clinical details and images.

The inclusion criteria consisted of confirmed periprosthetic joint infection with associated soft tissue defects requiring reconstructive intervention. All cases were managed with a multidisciplinary orthoplastic pathway involving orthopaedic surgeons, plastic surgeons, and infectious disease experts. Surgical management included radical debridement, implant removal when indicated, and placement of antibiotic-loaded cement spacers. Spacer design and antibiotic composition were individualized based on bone loss severity and microbial profile. Soft tissue reconstruction strategies included primary closure, local pedicle flaps, and medial gastrocnemius muscle flaps, selected according to defect characteristics, vascular status, and prior surgical history.

Intra-operative tissue samples were collected for microbiological analysis. Post-operative antimicrobial therapy was directed by infectious disease specialists and tailored to culture sensitivities, with intravenous therapy administered for 6–8 weeks, followed by an antibiotic-free interval prior to re-implantation where appropriate.

### 2.1. Patient Selection

Five patients who underwent surgery for infected knee arthroplasty associated with soft tissue defects were included.

The inclusion criteria included patients presenting with periprosthetic joint infection (PJI) following total knee arthroplasty, with concurrent soft tissue defects necessitating spacer placement and reconstructive soft tissue coverage, either primary closure, local pedicle flap, or muscle flap reconstruction.

The exclusion criteria included patients with simple PJIs without any requirement for plastic reconstruction or patient-specific spacers.

### 2.2. Data Collection

Clinical and surgical data were obtained from electronic medical records, operative notes, microbiology reports, and radiological imaging. The following variables were recorded:

Patient characteristics: demographics, comorbidities, smoking status, and alcohol use.

Index procedure: primary versus revision arthroplasty.

Infection characteristics: causative organisms, and infection chronicity.

Soft tissue reconstruction method: primary closure, local pedicle flap, medial gastrocnemius flap, bone reconstruction with spacer, sinus presence, and wound dehiscence.

Outcomes: infection eradication (absence of clinical or microbiological recurrence), functional range of motion (ROM), postoperative complications (wound breakdown, flap failure, reinfection), and follow-up duration.

### 2.3. Surgical and Multidisciplinary Approach

All cases underwent a multidisciplinary approach, including but not limited to orthopaedic, plastic, and infectious disease input. Patients underwent single-stage or two-stage revision procedures depending on infection chronicity, microbial profile, and host factors. Soft tissue management techniques included primary closure (n = 1), local pedicle flap (n = 1), medial gastrocnemius flap (n = 2), and a customised antibiotic-loaded cement spacer for bone defect (n = 1). Antimicrobial selection and duration were guided by intra-operative culture results and infectious diseases specialist consultation, with all patients receiving targeted intravenous antibiotic therapy postoperatively. Functional recovery was assessed by active and passive knee range of motion (ROM) measured clinically at follow-up visits, and ambulatory status at the final follow-up. Radiographic follow-up was performed at regular intervals to monitor implant position, fracture healing where applicable, and signs of recurrent infection. Patients were reviewed both clinically and radiographically at regular intervals throughout treatment for wound review, monitoring of inflammatory markers, and radiographic imaging.

Infection control was defined as a healed wound with no clinical signs of recurrence and normal biochemical markers (CRP/WCC/ESR). Functional outcomes were assessed through clinical examination involving range of mobility and ability to mobilise. Any data were anonymised, and patient consent for publication of anonymised clinical details and images was obtained.

### 2.4. Treatment Protocol

All patients were managed using a structured multidisciplinary orthoplastic protocol involving orthopaedic surgery, plastic and reconstructive surgery, and infectious diseases specialists. Management decisions were individualised based on infection chronicity, microbiological profile, soft tissue condition, bone loss, and host comorbidities; however, the overall treatment framework followed a consistent staged approach.

All patients underwent comprehensive clinical evaluation including assessment of wound status, presence of sinus tract, soft tissue viability, and neurovascular status. Laboratory investigations included inflammatory markers (CRP, ESR, WCC). Pre-operative imaging consisted of plain radiographs in anteroposterior and lateral views, with CT imaging where bone loss required further characterization. Joint aspiration was performed when feasible to guide microbiological diagnosis and antibiotic planning. Cases were discussed in a multidisciplinary setting to determine suitability for single-stage, two-stage, or modified (1.5-stage) revision.

All cases involved radical surgical debridement with removal of infected prosthetic components and excision of non-viable soft tissue. Multiple intra-operative deep tissue samples were obtained for microbiological culture and sensitivity testing. Antibiotic-loaded cement spacers were inserted in all staged cases. The spacer design (dynamic/articulating versus static/customized) was selected based on the degree of bone loss, soft tissue envelope integrity, need for joint stability, and dead-space management. Antibiotic selection and dosing within the cement were determined in consultation with infectious diseases specialists, guided by organism sensitivities and established dosing safety thresholds. Customised spacers were used in cases with significant bone defects to optimise mechanical stability, maintain limb length and alignment, and facilitate later preimplantation.

Soft tissue management was tailored according to defect size, location, and local tissue quality, using primary closure where feasible, local axial bi-pedicled flap for sinus control and moderate defects, and medial gastrocnemius muscle flap (with or without split-thickness skin graft) for larger anterior or parapatellar defects. Reconstruction was performed either during the first-stage procedure or in a coordinated staged manner depending on infection severity and wound condition.

Postoperatively, patients received culture-directed intravenous antibiotic therapy for 6–8 weeks under infectious diseases supervision. Following the completion of intravenous therapy, patients undergoing two-stage revision underwent an antibiotic-free interval prior to reassessment of inflammatory markers, clinical status, and, where indicated, repeat aspiration before second-stage preimplantation. In selected high-risk patients, long-term suppressive oral antimicrobial therapy was continued following reimplantation.

Second-stage preimplantation was performed once the clinical signs of infection had resolved, inflammatory markers had normalized or significantly improved, and wound healing was satisfactory. Definitive reconstruction included revision TKA components, metal augments, or distal femoral replacement where bone loss necessitated.

Patients were reviewed at regular intervals postoperatively with clinical wound assessment, measurement of range of motion (ROM) using clinical goniometry, assessment of ambulatory status, monitoring of inflammatory markers, and serial radiographs to assess implant position and detect recurrence. Infection control was defined as the absence of clinical recurrence and normalisation or stabilization of inflammatory markers at the latest follow-up.

## 3. Results

### 3.1. Summary of Patient Characteristics and Outcomes

Table 1 provides summary of patient characteristics and outcomes to clearly outline patient results.

### 3.2. Individual Case Summaries

Between 2023 and 2025, five patients (four male and one female; mean age of 68 years, range 51–83) were treated at our institution for complex peri-prosthetic joint infection (PJI) following TKA, with associated soft tissue defects in most (4/5). All patients underwent revision knee arthroplasty requiring soft tissue and bone reconstruction. Infective organisms varied across cases, including Staphylococcus epidermidis, methicillin-sensitive Staphylococcus aureus (MSSA), coagulase-negative Staphylococcus, Candida albicans, and mixed Gram-negative species (Escherichia coli, proteus mirabilis). All patients received targeted intravenous antibiotics—guided by cultures and sensitivities—for 6–8 weeks postoperatively, followed by an antibiotic-free interval prior to second-stage revision arthroplasty in most cases or long-term suppressive antimicrobial therapy in chronic cases.

**Figure 1:**
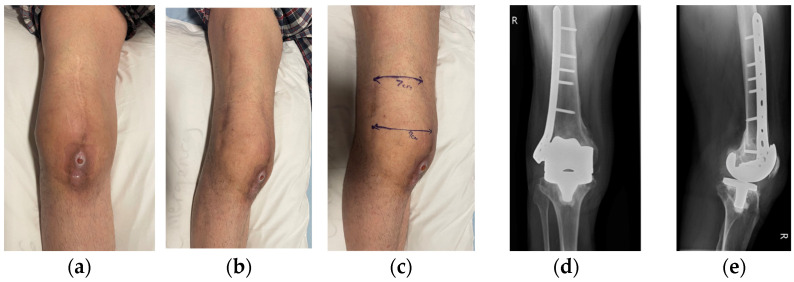
(**a**) Initial presentation of case 1 (anterior view). (**b**,**c**) Lateral view. (**d**) X-ray image from anterior view, and (**e**) X-ray image from lateral view.

Case 1 involved a 51-year-old male who presented with a stiff prosthetic knee (ROM 10–30°) and chronic sinus formation following right TKR in Ukraine complicated by a retained surgical swab and subsequent infection, as displayed in Figure 1. His background history was significant, including previous TKA followed shortly by a distal femoral peri-prosthetic fracture and fracture fixation (leading to a retained surgical swab), and longstanding common peroneal nerve palsy and foot drop. Intra-operative cultures grew coagulase-negative Staphylococcus and Staphylococcus epidermidis.

**Figure 2:**
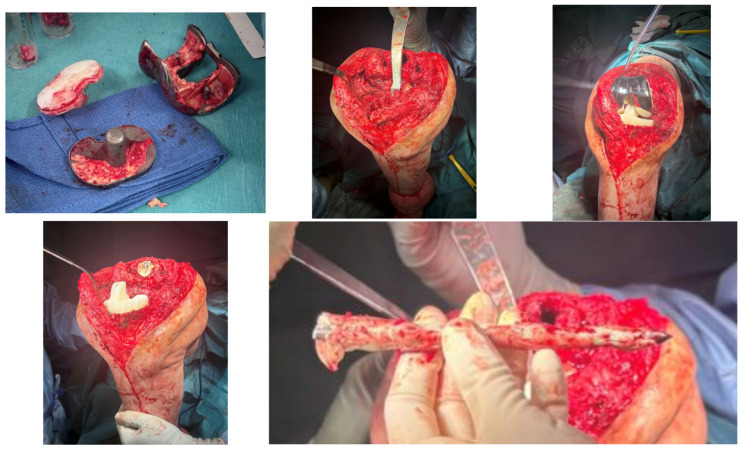
Intra-operative imaging of case 1 at first-stage revision.

**Figure 3:**
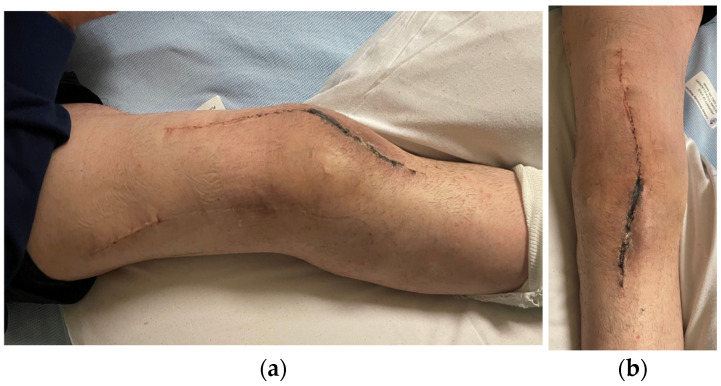
(**a**) Case 1 post-operative clinical findings (lateral view). (**b**) Anterior view of case 1 post-operatively.

**Figure 4:**
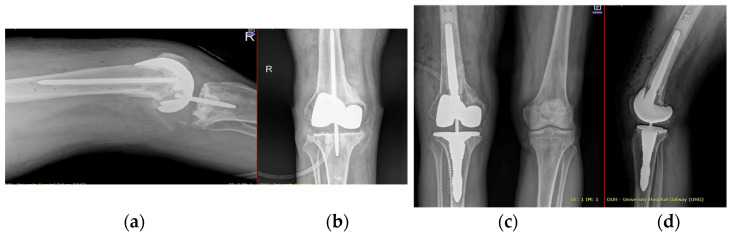
Radiographic findings demonstrating customized spacer and final implants at final follow-up of case 1. (**a**,**d**) Lateral view. (**b**,**c**) Posterior view. (**d**) Anterior view.

He underwent two-stage revision arthroplasty with extensive debridement and insertion of a dynamic antibiotic spacer (Figure 2), followed by re-implantation after a six-week antibiotic course followed by an antibiotic-free drug holiday. Soft tissue closure was achieved with primary closure in this case. At a twelve-month follow-up, the patient was pain-free and fully weight-bearing, with a range of motion (ROM) of 0–100 and complete wound healing (Figure 3). Imaging was satisfactory (Figure 4), and no recurrence of infection or further surgical intervention was required.

**Figure 5:**
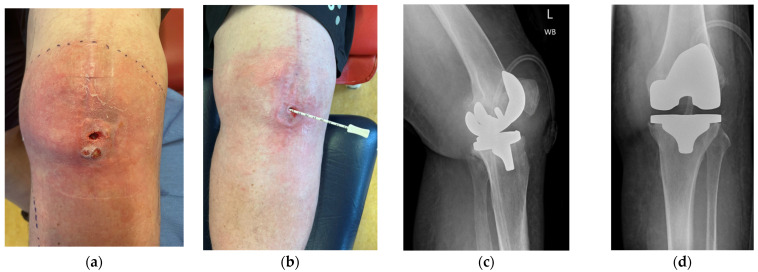
(**a**,**b**) Case 2 clinical findings at presentation and (**c**,**d**) radiographic findings during treatment.

**Figure 6:**
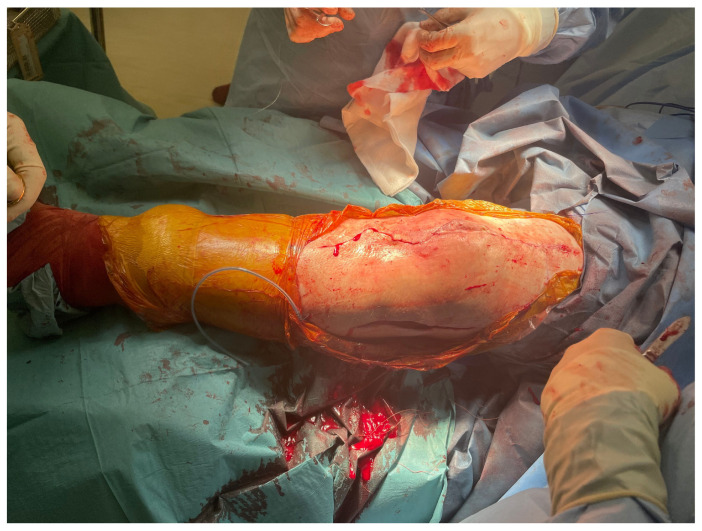
Case 2 intra-operative images of axial bi-pedicled flap for sinus control and soft tissue closure.

**Figure 7:**
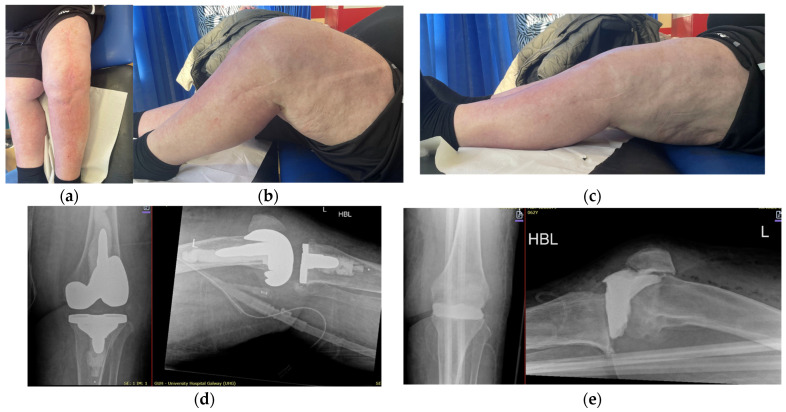
(**a**–**c**) Case 2 clinical findings at final follow-up. (**d**,**e**) Radiological imaging of spacer and revision prosthesis at final follow-up.

Case 2 was a 62-year-old male with significant comorbidities, including type 2 diabetes, thrombocytopenia, sarcoidosis, and cirrhosis secondary to Non-Alcoholic Fatty Liver Disease. This patient developed a left-knee PJI following elective TKA in May 2023 by a referring surgeon. Cultures obtained from the first stage of revision grew Pseudomonas aeruginosa, Klebsiella pneumoniae, and Staphylococcus epidermidis. He underwent a two-stage revision procedure, the first in January 2024, with the placement of an antibiotic spacer and soft tissue coverage using a local pedicle flap (Figure 5 and Figure 6). After six weeks of intravenous antibiotics, followed by an antibiotic-free interval, a second-stage re-implantation was completed in August 2024. The postoperative course was complicated by left-leg cellulitis (patient reported similar annual events prior to original TKA) but successfully managed with intravenous therapy with subsequent oral antimicrobial prophylaxis. At a one-year follow-up, the patient was infection-free, with a ROM of 0–95, ambulating independently with two crutches, and had fully healed soft tissue, as displayed in Figure 7.

**Figure 8:**
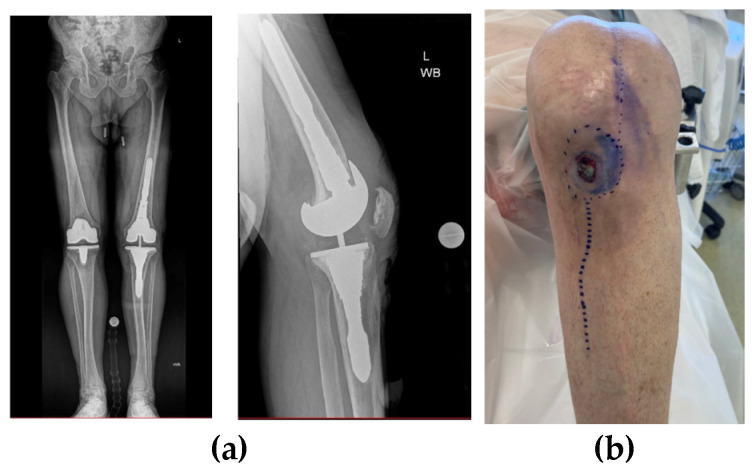
(**a**) Case 3 pre-op imaging. (**b**) Case 3 clinical findings at presentation.

**Figure 9:**
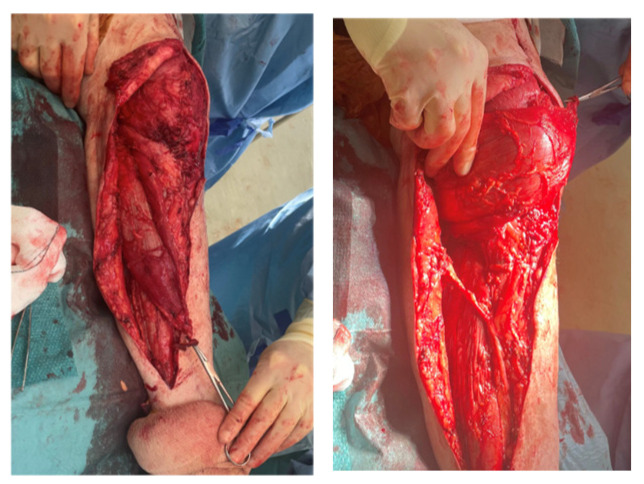
Case 3 intra-operative imaging demonstrating gastrocnemius flap elevation and coverage.

**Figure 10:**
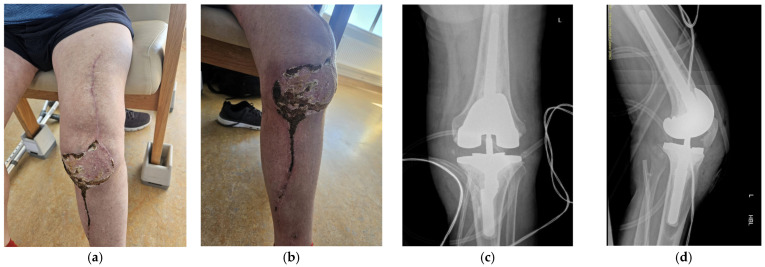
(**a**,**b**) Case 3 skin condition as of 10/04/25. (**c**,**d**) Plain films on 01/03/25 following second-stage revision.

Case 3 involved a 65-year-old male non-smoker with Parkinson’s disease who had a prior bilateral TKA (right, 2018; left, 2022) performed at another institution. This patient was treated with a two-stage revision TKA at the other institution, which failed and presented with a chronic discharging sinus and recurrent left-knee PJI (Figure 8). Cultures repeatedly grew Candida albicans, Staphylococcus epidermidis, and mixed anaerobes. A combined orthopaedic–plastic surgery revision procedure using a 1.5-stage implant technique was performed in February 2025, which consisted of a medial gastrocnemius flap coverage and skin graft (Figure 9). Intra-operative cultures confirmed polymicrobial infection, and the patient received six weeks of combination IV antibiotics followed by long-term oral suppression therapy. At a six-month follow-up, his wound was fully healed, donor site well epithelialized, and ROM 0–120 achieved (Figure 10). He remains infection-free and mobilising independently to date.

**Figure 11:**
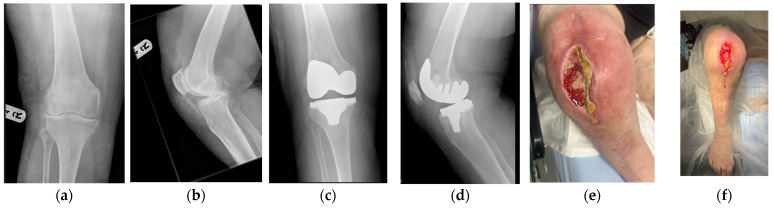
(**a**–**d**) Case 4 radiographic findings at presentation. (**e**,**f**) Case 4 clinical presentation.

**Figure 12:**
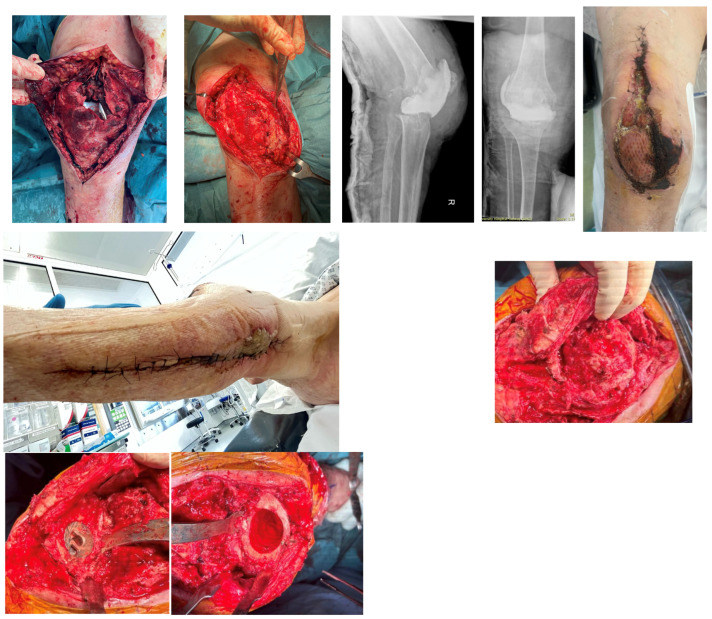
Case 4 throughout treatment course.

**Figure 13:**
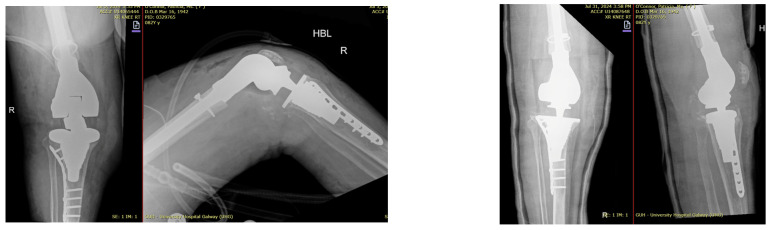
Post-op imaging for case 4 following second-stage revision.

Case 4 was an 83-year-old female who underwent right TKA at another institution in November 2023. Her past medical history was significant, including hypertension, atrial fibrillation, heart failure, peripheral vascular disease, and hypothyroidism. She re-presented in January 2024 with aggressive wound dehiscence and purulent drainage (Figure 11). Intra-operative cultures obtained during first-stage revision grew Escherichia coli and Proteus mirabilis. The patient underwent a two-stage revision TKA with medial gastrocnemius flap for soft tissue coverage (Figure 12). After eight weeks of antibiotics, she proceeded to the second-stage re-implantation in July 2024, utilising a distal femoral replacement due to intra-operative condyle fracture and poor bone stock (Figure 13). The post-operative course was complicated by a peri-prosthetic tibial fracture, treated with manipulation under anaesthesia and casting. Fracture union was achieved by mid-2025, approximately 18 months after initial presentation. At final follow-up, she was fully weight bearing with a rollator, had a ROM of 0–90 on clinical examination, and showed no recurrence of infection.

**Figure 14:**
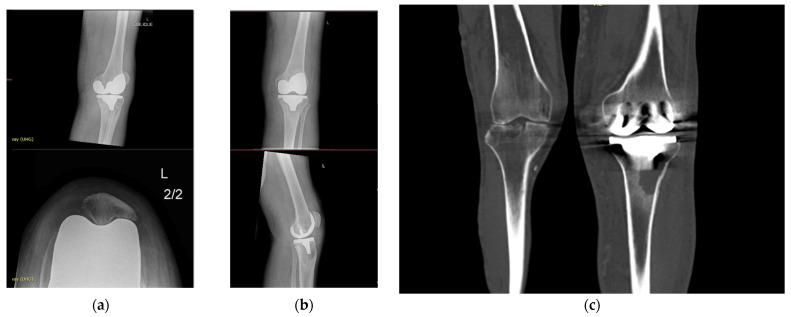
(**a**,**b**) Case 5 pre-op X-Ray. (**c**) CT demonstrating defect in tibia.

**Figure 15:**
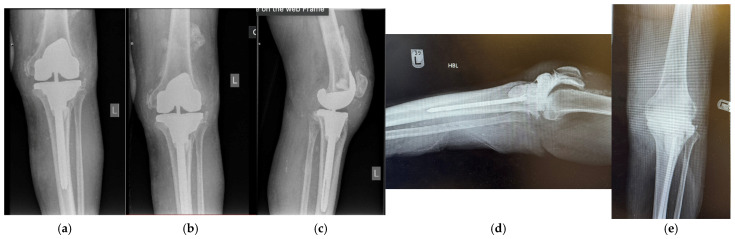
Post-operative X-rays for case 5: (**a**–**c**) spacer following first-stage revision and (**d**,**e**) radiographic findings upon completion.

Case 5 presented as a 79-year-old male with chronic left-knee infection one year after a primary left TKA carried out at another institution in October 2022. Cultures from joint aspiration grew Staphylococcus epidermidis, with significant tibial bone loss noted on imaging (AORI 2B defect, Figure 14). He underwent first-stage revision with removal of the infected prosthesis and placement of a customised static antibiotic-impregnated cement spacer. After completing six weeks of IV antibiotics and a six-week drug holiday, a culture-negative repeat aspiration deemed him suitable to proceed to second-stage re-implantation with the use of asymmetric metal augments in the tibia to accommodate the massive bone loss in April 2024. At an eight-month follow up, he was infection-free, with healed incisions, a ROM of 10–65, and no radiographic evidence of loosening or osteomyelitis (Figure 15).

### 3.3. Overall Outcomes

All cases achieved infection eradication, successful soft tissue coverage, and satisfactory functional recovery. All five patients were managed with multidisciplinary input and a personalized management approach, with no patient requiring amputation or re-operation for recurrent infection at the latest follow-up. Two patients remained on long-term antimicrobial suppression therapy for comorbidity-related risk factors. The gastrocnemius flaps (n = 2) and local pedicle flap (n = 1) all survived completely with no flap necrosis or wound breakdown. The customised spacer provided effective interim stability and infection control in the context of bone loss. All patients regained independent mobility and infection-free status at final review. However, while infection control was achieved in all cases, two patients remained on long-term suppressive antibiotic therapy at final follow-up, reflecting the complexity of infection management in this cohort.

## 4. Discussion

A key finding of this case series was that all five patients demonstrated infection control, functional restoration, and durable reconstruction, irrespective of the extent of soft tissue compromise or bone loss. The reconstructive strategies—ranging from primary closure to pedicled and gastrocnemius flaps—were selected based on defect characteristics and host factors, reflecting the importance of tailored reconstruction in infected TKA. No cases required amputation or revision beyond the staged procedures, and all achieved stable wound coverage with the restoration of functional ROM (mean 94, range 65–120). Importantly, all flaps survived, and all patients maintained ambulatory status. A tailored and heterogeneous approach was required to address the wide variety of clinical challenges in each specific case.

Infected TKA complicated by soft tissue loss is associated with increased risk of treatment failure and amputation [11,12]. Reported infection eradication rates after two-stage revision in such cases vary from 68 to 90%, depending on defect severity and host status [13,14]. The outcomes in this study—infection control and flap viability—are comparable or superior to published data, underscoring the benefit of early orthopaedic collaboration and tailored coverage selection. The use of medial gastrocnemius flaps for anterior or parapatellar defects remains the benchmark for reliable coverage [3]. Results from our retrospective case series reaffirm their effectiveness even in the context of recurrent or polymicrobial infection, as demonstrated in cases 3 and 4.

In this series, customised antibiotic-loaded cement spacers played a dual role in management, providing local infection control by maintaining high local antibiotic concentrations, and mechanical stability by preserving limb length, joint space, and soft tissue tension during the interim phase between debridement and re-implantation. Spacer customisation allowed the optimisation of joint stability, management of dead space following aggressive debridement, and maintenance of limb length and alignment, thereby facilitating subsequent soft tissue reconstruction and, where applicable, later preimplantation. These features were particularly relevant in cases with extensive bone loss of compromised soft tissue envelopes, where standard spaces may be insufficient. The customised static spacer used in case 5 provided temporary mechanical stability in the presence of extensive bone loss, which allowed for subsequent successful reconstruction. The functional benefits of spacer use in such cases have been consistently supported in the literature [11,13,15].

A central determinant of success was the multidisciplinary team (MDT) model, incorporating orthopaedic surgeons, plastics surgeons, and infectious disease specialists. Early joint decision-making allowed for optimal sequencing of debridement, coverage, and antimicrobial therapy. Infectious disease input was particularly critical in managing unusual organisms, including Candida albicans (case 3) and polymicrobial infections. This approach mirrors current recommendations for orthoplastic limb strategies in complex PJI [3,4,16,17,18].

The main limitations of this study include its retrospective design, small cohort size, single-centre bias, variable follow-up duration, and the absence of standardised patient-reported outcome measures, which may limit generalisability. Follow-up duration was not uniform across cases, and normal functional outcome scoring was not uniformly documented. Two individuals remained on suppressive antibiotic therapy at the latest review, which complicates the interpretation of long-term infection eradication. As such, the outcomes should be interpreted as short- to mid-term infection control rather than definitive cure. Functional outcomes were assessed by documenting the range of motion (ROM) measured clinically at follow-up visits. Formal patient-reported outcome measures were not consistently available across the cohort. In addition, the small sample size limits the feasibility of our results due to the heterogenous nature of patient outcomes. The marked heterogeneity in patient age, comorbidity burden, infecting organisms, bone loss, reconstructive strategies, and revision pathways reflects real-world complexity but precludes meaningful statistical comparison and limits the generalisability of conclusions. Despite these limitations, the qualitative consistency of infection control and wound healing outcomes strengthens the validity of our findings.

This series uniquely focuses on customisation and soft tissue management in the context of infected TKA—an area often underrepresented in the literature, which tends to emphasise the microbiological or prosthetic aspects of revision. It highlights the practical feasibility of combining standard two-stage revision principles with reconstructive adaptability supported by MDT collaboration. A key strength of this series is the early and integrated involvement of an orthoplastic multidisciplinary team, which allowed the simultaneous optimisation of infection control, skeletal reconstruction, and soft- tissue coverage—an approach which remains underreported in the existing literature.

## 5. Conclusions

This case series reinforces that infection control, functional limb salvage, and soft tissue reconstruction are achievable goals in complex revision TKA for PJI with soft tissue and bone defects when managed through an individualized, multidisciplinary approach. A tailored orthoplastic approach combining multidisciplinary planning, integration of custom antibiotic spacers, and timely flap coverage can achieve high rates of infection control and functional recovery, even in medically complex patients. This model is reproducible and may serve as a framework for managing similar high-risk cases in tertiary centres. Future prospective studies with larger cohorts are warranted to validate these findings and define optimal timing and sequencing of reconstructive interventions in infected TKA.

## Figures and Tables

**Table 1:** Individual case results demonstrating patient age, sex, soft tissue defect, reconstructive methods, spacer type used in each case, infective organism(s), and patient outcome.

Case	Age/Sex	Soft Tissue Defect	Reconstruction	Spacer Type	Organism(s)	Outcome
1	51 M	Chronic sinus	Primary closure	Dynamic spacer	Coagulase-negative strep Staphylococcus epidermis	Infection-free ROM 0–100
2	62 M	Sinus tract	Axial bi-pedicled flap	Articulating spacer	Pseudomonas, Klebsiella, Staphylococcus epidermis	Infection-free ROM 0–95
3	65 M	Chronic wound	Medial gastrocnemius flap	1.5-stage implant	Candida albicans, coagulase-negative strep, anaerobes	Infection-free ROM 0–120
4	83 F	Wound dehiscence	Medial gastrocnemius flap	Two-stage + distal femoral replacement	Escherichia coli, proteus	Infection-free ROM 0–90
5	79 M	Bone loss	Custom spacer	Custom	Staphylococcus epidermis	Infection-free ROM 10–65

## Data Availability

The raw data supporting the conclusions of this article will be made available by the authors on request.
